# Ultrastructure Expansion Microscopy applied to *C. elegans* embryos

**DOI:** 10.17912/micropub.biology.001033

**Published:** 2024-05-06

**Authors:** Valentin Burdet, Lorène Bournonville, Moushumi Das, Eva Wenger, Marie Delattre, Florian A. Steiner, Paul Guichard, Virginie Hamel

**Affiliations:** 1 Molecular and Cellular Biology Department, University of Geneva, Switzerland; 2 Ecole Normale Supérieure de Lyon, Laboratory of Biology and Modeling of the Cell, CNRS UMR5239, Inserm U1293, University Claude Bernard Lyon 1, 69007 Lyon, France

## Abstract

Visualization of organelles using expansion microscopy has been previously applied to *Caenorhadbitis elegans* adult gonads or worms. However, its application to embryos has remained a challenge due to the protective eggshell barrier. Here, by combining freeze-cracking and ultrastructure expansion microscopy (U-ExM), we demonstrate a four-time isotropic expansion of *C. elegans* embryos. As an example structure, we chose the nuclear pore and demonstrate that we achieve sufficient resolution to distinguish them individually. Our work provides proof of principle for U-ExM in *C. elegans* embryos, which will be applicable for imaging a wide range of cellular structures in this model system.

**Figure 1. U-ExM applied to C. elegans embryos:**
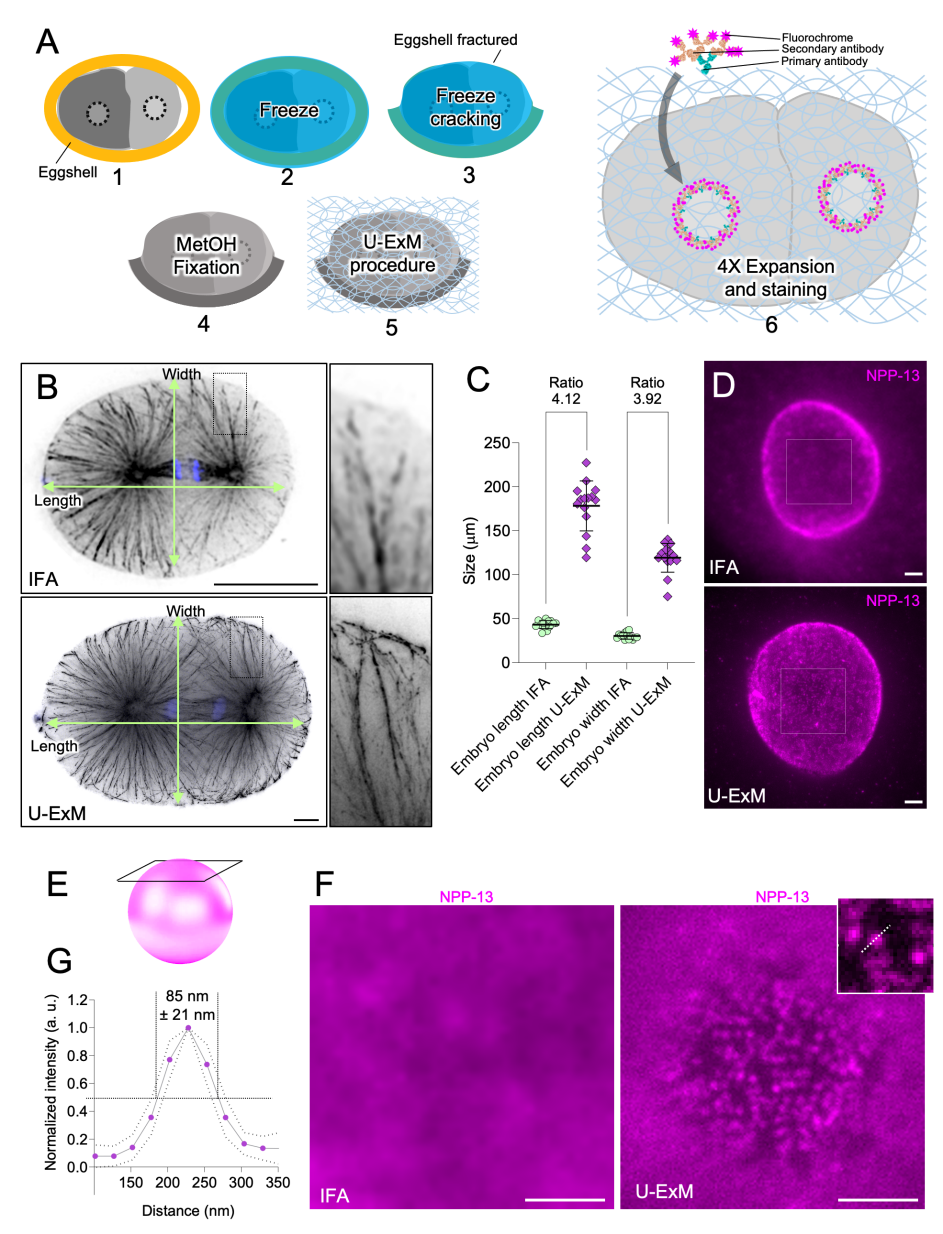
( **A** ) Schematic workflow depicting the different steps to perform U-ExM on *C. elegans* embryos including freeze-cracking (steps 1-3), fixation (steps 4), U-ExM procedure (step 5), and staining of the embryo post-expansion (step 6). ( **B** ) Representative widefield images of *C. elegans* one-cell stage embryos stained with rabbit a-tubulin antibody to visualize the microtubule network and the cell boundaries (top, without expansion; bottom, with expansion) and Hoechst to mark the DNA. The areas in the rectangular boxes are magnified on the right. IFA corresponds to conventional immunofluorescence and U-ExM to the expanded sample. Scale bars: 15 μm. The scale bar indicates actual measurements that have not been rescaled based on the expansion factor. ( **C** ) Measurements of the average width and length of the entire *C. elegans* embryo before and after expansion. Before expansion: w= 30.4 μm +/- 3.7 µm, L= 43.3 μm +/- 4.6 µm, N= 14 embryos; after expansion: w= 119.4 μm +/- 16.5 µm, L= 178.4 μm +/- 28.4 µm, N= 15 embryos; from 3 independent experiments. ( **D** ) Representative widefield images of *C. elegans* embryos nucleus stained with NPP-13 antibodies (magenta) to visualize nuclear pore complexes (top, without expansion; bottom, with expansion). IFA corresponds to conventional immunofluorescence and U-ExM to the expanded sample. Scale bars: 1 μm. The scale bar has been rescaled based on the expansion factor. ( **E** ) Schematic representation of region imaged to visualize the nuclear pores. ( **F** ) Enlargements of the dashed boxes in ( **D** ) within the region indicated in ( **E** ) highlighting NPCs that appear as individual dots after expansion. Images correspond to one Z-slice. Scale bar: 1 μm. The scale bar has been rescaled based on the expansion factor. ( **G** ) Quantification of the average width of NPP-13 dots after expansion. Widths were measured using a line plot profile as shown in the inset in ( **F** ). (N= 153, AVG= 85 nm +/- 21.4 nm, from 3 independent experiments).

## Description

Although expansion microscopy has been applied to *C. elegans* , it is not yet amenable to the egg-shelled *C. elegans* embryos, whose protection impedes proper expansion of this specialized developmental stage (Yu et al, 2020) . To circumvent this limitation, we undertook to combine the process of freeze-cracking, almost universally used to perform immunofluorescence microscopy of methanol-fixed *C. elegans* embryos, with the process of ultrastructure expansion microscopy (U-ExM) ( **Figure 1A** ) (Gambarotto et al, 2019; Duerr, 2006) . The freeze-cracking procedure aims to break off part of the eggshell by freezing the embryos between a glass slide and a coverslip and then rapidly flicking off the coverslip (Gönczy et al, 1999b). This makes the cells accessible for fixatives and antibodies. Briefly, young adult worms are deposited on a polylysine-coated glass slide and are cut open to release the embryos (see material and methods). After careful removal of the excess liquid, a 15-mm square coverslip is deposited on top, and the glass slide with the coverslip is moved onto a metal block cooled on dry ice. This instantaneously freezes the embryos and the liquid between the glass slide and the coverslip. After 10 min (or more), flicking off the coverslip cracks and removes part of the eggshell ( **Figure 1A** , steps 1 to 3). The glass slides with the cracked embryos are next plunged into -20°C methanol for fixation ( **Figure 1A** , step 4). Instead of proceeding with a regular immunofluorescence protocol (IFA), we apply the U-ExM protocol (Gambarotto et al, 2019, 2021) ( **Figure 1A** , step 5). After several washes in PBS, the slides are briefly dried. An anchoring solution containing 1.4% formaldehyde (FA) and 2% acrylamide (AA) is placed on top of the sample surrounded by a 0.3 mm thick spacer to contain the solution in place and then incubated overnight at 37°C before the gelation step. The samples are then further incubated with the non-activated monomer solution (19% sodium acrylate, 0.1% bis-acrylamide, and 10% acrylamide) for 15 min on a cold metallic block (placed on ice) to allow penetration of the solution in the sample. Subsequently, samples are treated with the activated monomer solution supplemented with TEMED and APS (final concentration of 0.5%) and covered with a 12-mm round coverslip for 30 min to 1 h at 37°C for the polymerization. Next, samples are denatured at 95°C for 1 h 30 min and the obtained gels are stained post-expansion (Gambarotto et al, 2019) before full expansion in pure water ( **Figure 1A,** step 6). The microtubule network and the cell boundaries of the embryos are visualized using tubulin antibodies, allowing monitoring of the expansion factor ( **Figure 1B** ). To that end, we measure the width and length of *C. elegans* two-cell stage embryos, as their dimensions are fixed, around 30 μm wide and 45 μm long. We demonstrate that embryos are expanded about 4-fold on average (before expansion (IFA): width= 30.4 μm +/- 3.7 µm, length= 43.3 μm +/- 4.6 µm; after expansion (U-ExM): width= 119.4 μm +/- 16.5 µm, length= 178.4 μm +/- 28.4 µm), indicating that the *C. elegans* embryos are overall isotopically expanded ( **Figure 1C** ). In addition, the gain in resolution brought by expansion microscopy is illustrated by the conservation of the elaborated microtubule network and the ability to resolve individual microtubules that are not distinguishable in classical immunofluorescence ( **Figure 1B** , insets).

To further assess the resolutive power of U-ExM applied to *C. elegans* embryos, we next turned to analyzing nuclear pore complexes (NPCs). NPCs are a hallmark of animal cells and are part of the nuclear envelope that separates the cytoplasm and the nuclear compartment. They are considered as the gatekeepers of the nucleus and regulate the exchange of macromolecules between these two compartments in interphase. NPCs are among the largest conserved proteinaceous assemblies in the cell. They consist of multiple copies of nucleoporins, which display a typical ring-like structure organization with an 8-fold radial symmetry (Beck & Hurt, 2017) . Homologs of nucleoporins have been identified in *C. elegans* and are referred to as NPP (Galy et al, 2003; Ródenas et al, 2012). NPP-13 , the homolog of vertebrate Nup93, is an inner nucleoporin component, which forms a subcomplex with NPP-3 /NUP205 and has been found to regulate NPC size exclusion, timing of mitotic entry and spindle orientation (Galy et al, 2003; Hawryluk-Gara et al, 2005; Schetter et al, 2006; Hachet et al, 2012) .

In *C. elegans* , NPCs have been visualized as a rim around the nuclear envelope using live cell microscopy as well as immunofluorescence microscopy ( **Figure 1D** ) (Lancaster et al, 2022; de la Cruz Ruiz et al, 2022) , but the resolution limit of such optical microscopy methods cannot resolve individual NPCs. We therefore decided to test, as a proof of principle, whether expansion microscopy would allow visualizing individual NPCs distributed on the nuclear envelope. Through conventional immunofluorescence (IFA), we confirmed that the nucleoporin NPP-13 signal exhibited a uniform rim-like pattern decorating the nuclear envelope, consistent with previous observations ( **Figure 1D** ) (Ferreira et al, 2017; Galy et al, 2003; Askjaer et al, 2014) . In contrast, by imaging the surface of the nucleus ( **Figure 1E** ), our expanded *C. elegans* embryos revealed individual dots scattered across the nuclear envelope, which likely correspond to individual NPCs ( **Figure 1E -F** and **Extended Data 1** ). Our analysis indicates that the signal width corresponding to individual NPP-13 dots averaged at 85 nm +/- 21 (N= 153 NPP-13 signals from 3 independent experiments) ( **Figure 1G** ), a dimension that aligns with the average width of NPC in tangential views measured at 75.4 nm +/- 8.4 in transmission electron microscopy (Cohen, 2002) . This resolution is attained through two key factors: first, the expansion factor, and second, the size of the antibodies relative to the target proteins, which decreases proportionally with the expansion factor, consequently reducing linkage errors and enhancing localization accuracy (Hamel & Guichard, 2021) .

In summary, we have successfully overcome the challenges posed by the egg-shelled *C. elegans* embryos to the application of expansion microscopy, by combining freeze-cracking and U-ExM to achieve a four-fold isotropic expansion. This expansion, which preserves the ultrastructure of the embryos, as demonstrated by the intact microtubule cytoskeleton network, enabled us to resolve single microtubules as well as individual nuclear pore complexes (NPCs) within these embryos using widefield light microscopy, a feat typically attainable only through super-resolution or electron microscopy techniques. Our findings highlight the versatility and applicability of U-ExM in visualizing protein complexes within the *C. elegans* embryo, thus enhancing the existing arsenal of cell biology tools for this model system.

## Methods

***C. elegans* strains and maintenance**

The N2 Bristol strain ( *C. elegans* wild isolate) was used in this study. The worms were maintained on *E. coli* OP50-seeded Nematode Growth Medium (NGM) plates at 20°C according to standard procedures.

**Freeze-cracking procedure followed by indirect immunofluorescence (IFA)**

Indirect immunofluorescence was performed as previously described (Gönczy et al, 1999a). Briefly, slides were coated with poly-L-lysine (2 mg/ml in water) and briefly heated at 200°C for polymerization, just before usage. Then, 8-10 μl of a mix composed of 50% water and 50% M9 buffer was pipetted in the center of the coated slide and 20-30 young adult hermaphrodites were deposited and cut open with a scalpel to release the embryos. Note that worms should be concentrated in the center of the slide to facilitate the subsequent steps of the protocol. After placing a 15-mm x 15-mm square coverslip on top of the worms and embryos, the excess liquid was carefully and quickly removed using a piece of Whatman 3MM paper if needed. This step was monitored under a dissecting microscope, enabling to stop when the embryos were flattened without exploding. The slide was next quickly transferred onto a pre-cooled metal block on dry ice and pressed down. After a minimum of 10 min, the coverslips were flicked off with a razor blade and the slides were immediately plunged into precooled -20°C methanol for 5 min. Slides were then immersed in PBS for 5 min two times before being incubated with 2% PBS-BSA for 20 min at room temperature and subsequently incubated with primary antibody mix (rabbit α-tubulin 1/1000 in PBS, or guinea pig α-tubulin and β-tubulin antibodies 1/500 each in PBS-BSA 2%, or anti- NPP-13 1/500 in PBS/BSA 2%) overnight at 4°C in a humid chamber. After three washes in PBS, slides were incubated with the following secondary antibodies: 1/1000 anti-rabbit-Cy3 in PBS or 1/800 anti-rabbit-Alexa488 in PBS/BSA 2% or 1/800 anti-guinea pig-Alexa568 in PBS/BSA 2%, and Hoechst 1/1000 for 1 h at room temperature in the dark. Final washes were done in PBS for 5 min. Slides were mounted in one drop of mounting medium (4% n-propyl gallate and 90% glycerol in PBS). A square 15-mm coverslip was placed onto the sample and sealed with nail polish before imaging or storage at 4°C.

**U-ExM**

After freeze-cracking, the slides containing the embryos were methanol-fixed as indicated above. After transferring the slides into PBS, the U-ExM protocol was performed as described (Gambarotto et al, 2019, 2021) . Briefly, round spacers of 0.3 mm thickness (IS317, SunJin Lab Co) were stuck around the samples on the slides and then, 300 μl of the anchoring solution (2% AA + 1.4% FA diluted in PBS) was added, contained by the spacers. The slides were incubated overnight at 37°C. Next, the AA/FA solution was removed, and 90 μl of monomer solution (not activated, without TEMED and APS) was added, still contained by the spacers. The slides were incubated for 15 min on a cold metallic block. Then, the spacers were removed and the monomer solution (19% sodium acrylate, 0.1% bis-acrylamide, and 10% acrylamide) supplemented with TEMED and APS (final concentration of 0.5%) was placed on top of the sample (35 μl final volume), which was then covered with a 12-mm round coverslip to initiate the gelation. The slides were incubated for 15 min on a cold metallic block and then for 30 min to 1 h at 37°C in a humid chamber. After the gelation, slides were submerged in denaturation buffer (200 mM SDS, 200 mM NaCl, and 50 mM Tris in nuclease-free water, pH 9) and placed on a shaker (60 rpm) at room temperature for 15 min to let the gels detach from the slides and the coverslips. The gels were then carefully removed from the slides and the coverslips and next placed in individual 1.5-ml micro-centrifuge tubes containing 1 ml of denaturation buffer. Denaturation was performed for 1 h 30 min at 95°C followed by 3 washes of at least 30 min each in ddH _2_ O, using either a beaker or a 10-cm petri dish. The gels were next measured with a ruler to calculate the expansion factor by dividing the final size of the gel after expansion by the initial dimension of 12 mm. Measuring the expansion factor of the gel is a pre-requisite to ensure that the overall expansion protocol was correctly performed. After this step, the edges of the expanded gels were re-cut with a razor blade to fit inside a 6-well plate while checking that the sample was in the center of the gel using a basic stereomicroscope. Then, the gels were shrunk using 3 baths of PBS of 10 min each to fit them in a 12-well plate, before incubation with 500 μl of antibody solutions. Gels were stained using the following primary antibodies in PBS-BSA 2% overnight at 4°C: rabbit α-tubulin antibody 1/500 or a mix of guinea pig α-tubulin and β-tubulin antibodies 1/250 each or NPP-13 1/250. On the next day, the gels were washed in PBS-Tween 0.1 % 3 times at room temperature on agitation (80 rpm), and then were incubated with secondary antibodies in PBS-BSA 2% (1/500 anti-rabbit-Cy3 or 1/400 anti-rabbit-Alexa488 or 1/400 anti-guinea pig-Alexa568) and Hoechst 1/1000 for 2.5 h at 37°C under agitation (80 rpm). Finally, 3 washes in PBS-Tween 0.1 % at room temperature on agitation (80 rpm) were performed and then the gels were fully expanded with 3x 10 min washes in ddH _2_ O in a 6-well plate.

**Imaging**

Expanded gels were placed onto 24 mm coverslips coated with poly-D-lysine (0.1 mg/ml) and imaged with an inverted widefield Leica DM8 Thunder microscope using a 63x 1.4NA oil immersion objective. Images were taken with a 63x 1.4 NA oil objective using the Thunder “Small volume computational clearing” mode and water as “mounting medium” to generate deconvolved images. 3D stacks were acquired with 0.21 µm z-intervals and a 100 nm x, y pixel size. For the classical immunofluorescence, images were acquired with the same inverted widefield Leica DM8 Thunder microscope using a 63x 1.4NA oil immersion objective.

**Visualization, quantification, and statistical analysis**

Visualization and image analysis were done using Fiji (ImageJ version 1.53f51) (Schindelin et al, 2012) . The immunofluorescence images in Figure 1D correspond to the maximum intensity projection of the upper half of the nuclei . Measurements of the *C. elegans* embryos by classical immunofluorescence or U-ExM were done manually using the *Straight* tool to measure the length and width of the embryos through their center using the tubulin labeling, which enabled us to identify the embryo shapes.

For measurements of the nuclear pore complexes sizes stained with the nucleoporin NPP-13 in U-ExM, the Fiji plot profile tool was used to obtain the fluorescence intensity profile from individual NPP-13 dots.

**Troubleshooting**

The primary limitation we encountered was related to the working distance constraint of the microscope when imaging *C. elegans* embryos. As the embryo became four times bigger in all dimensions after expansion, the working distance of the objective became a limiting factor. To address this issue, we recommend using a water objective. Furthermore, we observed that achieving a successful freeze-cracking of the eggshell before expansion is crucial for obtaining high-quality images. Inadequate cracking can lead to partial or poor antibody staining in both classical immunofluorescence and expansion microscopy protocols.

## Reagents

**Table:** 

**Product**	**Supplier**	**Reference**
Acrylamide 40% w/w	Merck – Sigma Aldrich	A4058
Formaldehyde 35-38%	Merck – Sigma Aldrich	F8775
Sodium acrylate	AK Scientific	R624
Ammonium persulfate (APS)	Thermo Fisher	17874
Tetramethylethylenediamine (TEMED)	Thermo Fisher	17919
Poly-D-Lysine	Thermo Fisher	A38904-01
Poly-L-Lysine	Merck – Sigma Aldrich	P1524
Bovine Serum Albumin (BSA)	Merck – Sigma Aldrich	10735086001
Tween 20	Roth	9127-2
Sodium dodecyl sulfate (SDS)	PanReac AppliChem	A2572
Sodium Chloride (NaCl)	Roth	0601.2
Tris-Base	Roth	2449.3
Rabbit α Tubulin polyclonal	Abcam	18251
Guinea pig α Tubulin monobody	ABCD antibody	AA344
Guinea pig β Tubulin monobody	ABCD antibody	AA345
Rabbit NPP-13 polyclonal antibody	Hachet et al, 2012	
Hoechst – BisBenzimid H 33258	Merck – Sigma Aldrich	B2883
Rabbit-Alexa488	Invitrogen	A11008
Guinea Pig-Alexa568	Invitrogen	A11075
Rabbit-Cy3	Jackson Immuno Research	711-165-152
Mounting medium	Vector laboratories	H-1000

## Extended Data

Description: Extended data 1. Individual nuclear pores in the C. elegans embryo visualized using U-ExM.. Resource Type: Audiovisual. DOI: 10.22002/5bjrg-98107
